# Performance of Calcium Phosphate Cements in the Augmentation of Sheep Vertebrae—An Ex Vivo Study

**DOI:** 10.3390/ma14143873

**Published:** 2021-07-12

**Authors:** Raimund W. Kinne, Francesca Gunnella, Elke Kunisch, Sascha Heinemann, Berthold Nies, Stefan Maenz, Victoria Horbert, Bernhard Illerhaus, René Huber, Izabela Firkowska-Boden, Jörg Bossert, Klaus D. Jandt, André Sachse, Matthias Bungartz, Olaf Brinkmann

**Affiliations:** 1 Experimental Rheumatology Unit, Department of Orthopedics, Jena University Hospital, Waldkliniken Eisenberg GmbH, 07607 Eisenberg, Germany; fgunnella@alice.it (F.G.); elke.kunisch@med.uni-heidelberg.de (E.K.); victoria.horbert@med.uni-jena.de (V.H.); a.sachse@waldkliniken-eisenberg.de (A.S.); m.bungartz@krankenhaus-eisenberg.de (M.B.); drbrinkmann@t-online.de (O.B.); 2 INNOTERE GmbH, Meissner Str. 191, 01445 Radebeul, Germany; S.Heinemann@innotere.de (S.H.); berthold.nies@innotere.de (B.N.); 3 Chair of Materials Science, Otto Schott Institute of Materials Research, Friedrich Schiller University Jena, 07743 Jena, Germany; stefanmaenz@aol.com (S.M.); izabela.firkowska-boden@uni-jena.de (I.F.-B.); joerg.bossert@uni-jena.de (J.B.); k.jandt@uni-jena.de (K.D.J.); 4 Jena Center for Soft Matter (JCSM), Friedrich Schiller University Jena, Humboldtstr. 10, 07743 Jena, Germany; 5 BAM Bundesanstalt für Materialforschung und –Prüfung (BAM), 12205 Berlin, Germany; illerhaus@berlin.de; 6 Institute of Clinical Chemistry, Hannover Medical School, 30625 Hannover, Germany; huber.rene@mh-hannover.de; 7 Jena School for Microbial Communication (JSMC), Friedrich Schiller University Jena, Neugasse 23, 07743 Jena, Germany; 8 Orthopedic Professorship, Department of Orthopedics, Jena University Hospital, Waldkliniken Eisenberg GmbH, 07607 Eisenberg, Germany

**Keywords:** sheep, PMMA, calcium phosphate bone cement, oil-based, ready-to-use, water-based, micro-CT, compressive strength, Young’s modulus

## Abstract

Oil-based calcium phosphate cement (Paste-CPC) shows not only prolonged shelf life and injection times, but also improved cohesion and reproducibility during application, while retaining the advantages of fast setting, mechanical strength, and biocompatibility. In addition, poly(L-lactide-co-glycolide) (PLGA) fiber reinforcement may decrease the risk for local extrusion. Bone defects (diameter 5 mm; depth 15 mm) generated ex vivo in lumbar (L) spines of female Merino sheep (2–4 years) were augmented using: (i) water-based CPC with 10% PLGA fiber reinforcement (L3); (ii) Paste-CPC (L4); or (iii) clinically established polymethylmethacrylate (PMMA) bone cement (L5). Untouched (L1) and empty vertebrae (L2) served as controls. Cement performance was analyzed using micro-computed tomography, histology, and biomechanical testing. Extrusion was comparable for Paste-CPC(-PLGA) and PMMA, but significantly lower for CPC + PLGA. Compressive strength and Young’s modulus were similar for Paste-CPC and PMMA, but significantly higher compared to those for empty defects and/or CPC + PLGA. Expectedly, all experimental groups showed significantly or numerically lower compressive strength and Young’s modulus than those of untouched controls. Ready-to-use Paste-CPC demonstrates a performance similar to that of PMMA, but improved biomechanics compared to those of water-based CPC + PLGA, expanding the therapeutic arsenal for bone defects. O, significantly lower extrusion of CPC + PLGA fibers into adjacent lumbar spongiosa may help to reduce the risk of local extrusion in spinal surgery.

## 1. Introduction

Bone cements are widely used in different applications of orthopedic surgery due to their injectability, especially for minimally invasive surgical techniques, such as vertebroplasty (VP) or kyphoplasty (KP; [1,2,3]). They are generally supplied as self-curing systems, consisting of separate powder and liquid phases, and mixed prior to use during surgery. The materials undergo in situ polymerization following injection into the vertebral body and harden to provide adequate mechanical support to the vertebral column [4]. Currently, the most popular are polymethylmethacrylate (PMMA)-based acrylic bone cements with high mechanical strength and successful use in VP or KP to provide pain relief and stability to the fracture site [1,2,3]. However, PMMA presents some potential drawbacks, including non-biodegradability, monomer toxicity, heat generation during exothermic polymerization (up to 60 °C), and higher stiffness than that of cancellous bone, all of which possibly contribute to fractures of adjacent vertebrae after VP [5,6,7]. To overcome these disadvantages, biodegradable calcium phosphate cements (CPC) were recently developed, which show excellent osteoconductivity, resorbability, and a setting reaction at body temperature. However, the handling process, short working times, and poor mechanical properties constitute major challenges toward their clinical application [8,9]. Because of the low mechanical strength, CPC is mainly used in non- or moderate load-bearing sites. The inclusion of fibers (non-degradable or biodegradable) into the CPC appears a viable option to improve its mechanical strength [9,10,11]. In a large animal model, a recently developed biodegradable CPC with poly(L-lactic-co-glycolic acid) (PLGA) fiber reinforcement demonstrated excellent biocompatibility and sufficient strength and/or stiffness required for minimally invasive bone augmentation in load-bearing areas [10,11,12,13]. The complex handling process and short working time, however, remain critical points for the reproducible preparation of a homogenous, injectable cement paste given the time pressure and different environmental conditions during surgery. A delay in the application of the cement or a prolongation of the operation time, for example, may lead to contamination of the material and increased risk of infections [14]. To overcome such drawbacks, so-called ready-to-use (pre-mixed) cements were developed [15,16]. In this formulation, the cement powder is dispersed in a water-miscible solvent, and the hardening occurs by diffusion of tissue water into the cement paste only after injection into bone defects. Recently, the combination of a water-immiscible carrier liquid (e.g., synthetic short-chain triglyceride) with surfactants was shown to facilitate a discontinuous liquid exchange in the CPC, leading to an improvement in the shelf life of the pre-mixed paste and a higher reproducibility during application and setting reaction [16].

This novel paste CPC retained the well-known biocompatibility of water-based CPC, as shown by: (i) absence of in vitro cytotoxicity in standardized tests with unreacted Paste-CPC; and (ii) lack of sensitization, intracutaneous reactivity, and systemic toxicity in animal studies. The biocompatibility of the Paste-CPC was also confirmed in animal tests showing reactions similar to those of a commercial reference CPC and, finally, by the proliferation and differentiation of human mesenchymal stem cells into osteoblasts on plotted Paste-CPC samples ([16] and references therein).

Given the different biomechanical properties of bones in the human body, it is highly desirable to have multiple options of tailored bone-filling materials. Thus, the present study compared characteristics of marketed or newly developed bone-filling materials to provide a more systematic background for an evidence-based choice. In detail, the comparison focused on the effects of the ex vivo injection into sheep cadaver vertebral bodies of the ready-to-use oily cement Paste-CPC compared to both commercial PMMA cement and the newly developed fiber-reinforced CPC (CPC + PLGA), using micro-computed tomography (micro-CT), histology, and biomechanical tests.

A particular focus of the present study was the analysis of the extrusion of conventional CPC (+fibers) in comparison to that of Paste-CPC and PMMA cement immediately adjacent to the injection channel, including potential contributing factors, such as: (i) leakage through large vertebral veins or bone marrow channels opened by defect generation; (ii) for PMMA, tissue necrosis caused by high polymerization temperatures; (iii) choice of injection site, pressure, and cement volume; and (iv) different viscosities of CPCs and PMMA upon shear stress.

## 2. Materials and Methods

### 2.1. Preparation of Bone Cements

A mini extrusion system (RANDCASTLE EXTRUSION SYSTEMS INC., Cedar Grove, NJ, USA) was used to extrude PLGA fibers with a final diameter of 25 µm from granules (PURASORB PLG 1017; Purac, Gorinchem, The Netherlands). Fibers were subsequently cut to a length of 0.6 mm using a cutting mill (PULVERISETTE 19; FRITSCH GmbH, Idar-Oberstein, Germany) with a 1 mm sieve insert.

CPC powder of a commercial, water-based, brushite-forming bone cement was used (JectOS+; Kasios, L’Union, France; [12,13,17]). The CPC powder consisted of 81.6% (*w*/*w*) β-tricalcium phosphate (β-TCP); 16.7% (*w*/*w*) zirconium dioxide; and 1.7% (*w*/*w*) tetrasodium pyrophosphate, the liquid of an aqueous solution containing 3.0 M phosphoric acid and 0.1 M sulfuric acid [18]. In addition, 10% *w*/*w* PLGA fibers were added to the CPC powder (final powder-to-liquid proportion 2.2 g/mL) and homogenized for ≤2 min to obtain an injectable, paste-like consistency [10,11,12,13,17]. High-viscosity radiopaque PMMA cement (Kyphon HV-R, Medtronic Inc., Milan, Italy; powder-to-liquid proportion 2.1 g/mL) was hand-stirred using a spatula for 2 min and then injected. In addition, a ready-to-use, oil-based, hydroxyapatite-forming CPC cement was employed (Paste-CPC; INNOTERE GmbH, Radebeul, Germany; [16]). The paste (3 mL) was prepared as published and injected through a custom application cannula [16].

### 2.2. Cement Injection

**Figure 1:**
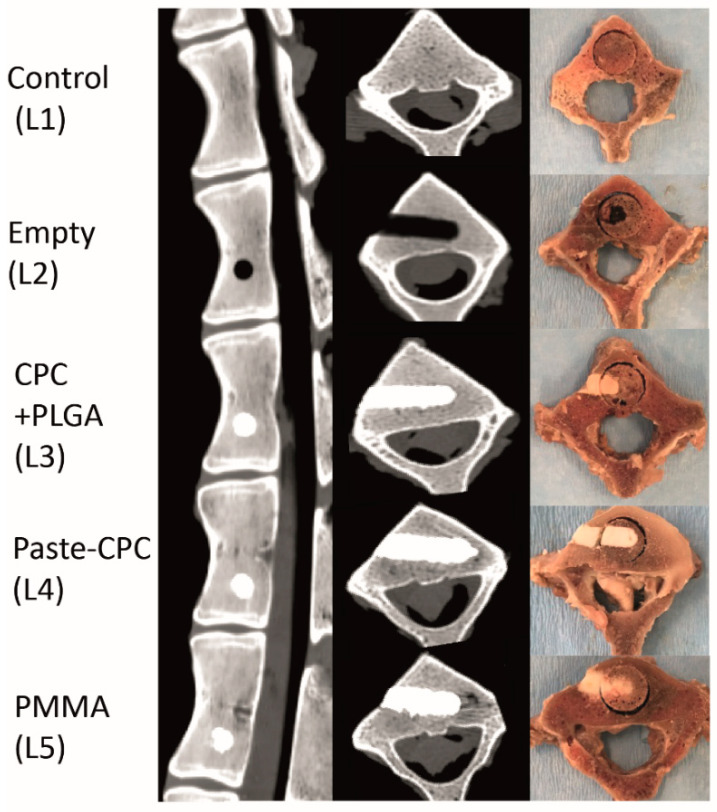
Depiction of cement localization by computed tomography (left—sagittal; middle—transversal) or gross morphology (right); L1 = untouched control; L2 = empty defect; L3 = CPC + PLGA; L4 = Paste-CPC; L5 = PMMA (Kyphon HV-R). CPC, calcium phosphate cement; PMMA, polymethylmethacrylate; PLGA, poly(L-lactide-co-glycolide). For scalebars please refer to Figure 2.

Ex vivo cement injection was performed using deep frozen spinal columns (lumbar vertebrae L1–L5) of 30 female Schwarzkopf–Merino sheep (age 3.0 ± 0.2 years, mean ± standard error of the mean (SEM); body weight of 65.8 ± 2.0 kg). Despite its quadruped gait, this large animal model is regarded as highly similar to the human situation [19,20] and may provide more homogeneous conditions than human samples when pre-grouped for gender, breed, age, and weight [20,21,22,23,24]. In addition, on the basis of a comparable structure of the thoracic and lumbar spine, the sheep spine may represent a useful experimental model for the augmentation of vertebral body defects with bone cement [20]. Animals operated as controls for unpublished studies of experimental osteochondral stifle joint repair were used (permission number 02-029/14; governmental commission for animal protection, Free State of Thuringia, Germany). After complete thawing, the paraspinal muscles were removed to permit a visually controlled cement injection. Bone defects were generated at room temperature in the lower third of the vertebral body by advancing a hand-guided surgical drill (5 mm in diameter; Stryker Leibinger GmbH, Freiburg, Germany) toward the center of the vertebral body. A final depth of the drill channel of approx. 15 mm was defined by an external marker tape. After removal of the drill, a maximum of 0.30 mL of the three cements was slowly injected under low-pressure conditions until complete filling of the defect using a separate bone filler/pestle system (Medtronic, Minneapolis, MN, USA) [12,17]. The following allocation was used: L1 = untouched (control); L2 = empty defect; L3 = CPC + PLGA; L4 = Paste-CPC; L5 = PMMA (Figure 1).

### 2.3. High-Resolution Micro-CT and Analysis

Individual vertebral bodies of 20 sheep were imaged three-dimensionally using an X-RAY WorkX 225 kV tube micro-CT system, a minimal spot size below 5 µm, a flat panel detector (PerkinElmer 1621; CsJ as scintillator; 2048 × 2048 pixel), and previously reported settings ([12,17]; final voxel resolution 66.6 µm). Quantitative analysis was performed with the 3D software VGSTUDIO MAX 2.2 (Volume Graphics GmbH, Heidelberg, Germany). Onion shell half-cylinders with radii of 2.5, 3, 3.5, 4, 4.5, and 5 mm were used for global threshold determination [12,13,17]. Cement volume/total volume is expressed as means ± SEM.

### 2.4. Biomechanical Testing (Compressive Strength)

For the biomechanical measurements of the compressive strength, the vertebral bodies from 10 sheep were initially prepared by removing the end plates (final height 15 mm) to obtain parallel surfaces for axial load application. Then, a trephine (diameter 10 mm) was used to extract frozen vertebral spongiosa cylinders (diameter 10 mm, height 15 mm; see Figure 1). The cylinders were thawed at room temperature for precisely 30 min and compressed along their longitudinal axis to determine failure load and Young’s modulus as previously published [17].

### 2.5. Histology

In order to assess the cement leakage into the adjacent spongiosa, the vertebral bodies L1–L5 of *n* = 3 animals were cut into two parts along the axis of the cement injection channel using a water-cooled diamond saw and then processed in different ways according to the different composition of the three cements [17].

The vertebral bodies injected with CPC were prepared for decalcified paraffin sections, whereas those injected with PMMA and Paste-CPC were embedded in 8% gelatin and processed for undecalcified cryostat sections as previously reported ([17] and references therein). The undecalcified cryostat sections were used for PMMA and Paste-CPC, because—possibly due to organic solvents—these bone cements dissolved with all other embedding and staining techniques [17]. Hematoxylin/eosin or hematoxylin staining was performed for all sections, and representative samples were then photographed.

### 2.6. Statistical Analyses

The results are displayed as means ± SEM. SPSS 22.0 software (SPSS Inc., IBM Corp. Armonk, NY, USA) was used to perform the statistical analyses. Paired samples were compared using the non-parametric Wilcoxon rank sum test (accepting *p* ≤ 0.05 as significant).

On the basis of a previous study comparing the extrusion of CPC reinforced with 10% poly(l-lactide-coglycolide) (PLGA) fibers or PMMA cement at 1.0, 1.5, 2.0, and 2.5 mm distance from the edge of the injection channel by micro-CT [17], the effect sizes between 1.0 and 1.23 were calculated, which is considerably higher than the medium effect size of 0.5. Using this effect size in a two-tailed Wilcoxon signed-rank test (matched pairs) with an α of 0.05 and a power of 0.80, sample numbers between 8 and 11 were computed (G*Power 3.1.9.7). To address a potentially lower difference between fiber-reinforced water-based CPC and Paste-CPC in the present study, the sample number was raised to *n* = 20 for micro-CT analysis and chosen at the higher end of the computed sample numbers (*n* = 10) for biomechanical testing.

Analyses of the micro-CT data and biomechanical testing were performed by one researcher each (S.M. and E.K., respectively), and both researchers were blinded to the experimental status of the samples.

## 3. Results

### 3.1. Micro-CT Analyses

**Figure 2:**
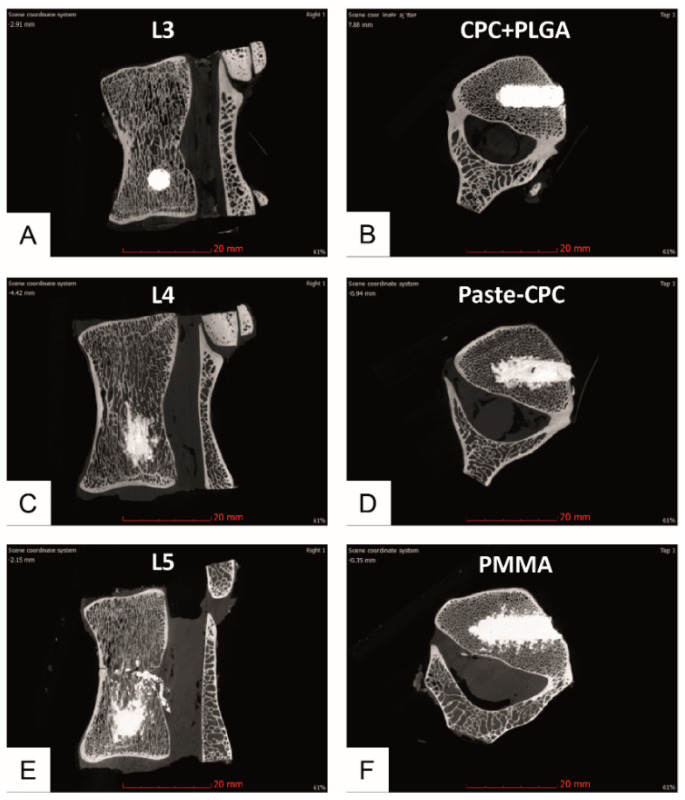
Representative micro-computed tomography (CT) pictures of vertebrae injected ex vivo with CPC + PLGA ((**A**)—sagittal; (**B**)—transversal), Paste-CPC (**C**,**D**), or PMMA (**E**,**F**); micro-CT images were quantitatively evaluated using half-cylinders of 2.5, 3, 3.5, 4, 4.5, and 5 mm radius; (**C**,**E**) exemplify cement extrusion via basivertebral veins; CPC, calcium phosphate cement; PLGA, poly(L-lactide-co-glycolide); PMMA, polymethylmethacrylate. Please note scalebars.

Conventional CPC + PLGA demonstrated clearly reduced leakage into the adjacent spongiosa marrow when compared to either Paste-CPC or PMMA (Figure 2).

**Figure 3:**
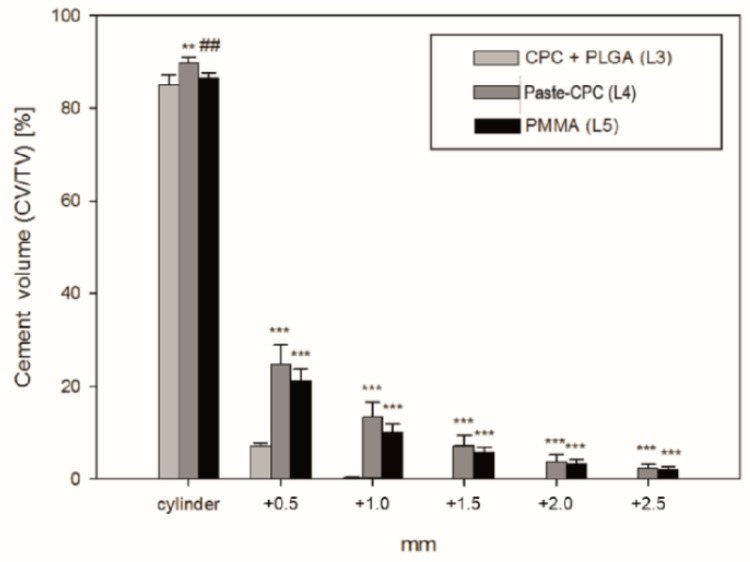
Quantitative analysis of micro-computed tomography pictures of vertebral bodies ex vivo injected with different cements; ** *p* ≤ 0.01, or *** *p* ≤ 0.001 versus CPC + PLGA (L3); ## *p* ≤ 0.01 versus Paste-CPC (L4); CPC, calcium phosphate cement; PLGA, poly(L-lactide-co-glycolide); PMMA, polymethylmethacrylate.

This was confirmed by quantitation of the cement extrusion, which noticeably decreased for all bone cements with increasing distance from the injection channel. However, the values for the CPC + PLGA were significantly lower compared to both Paste-CPC and PMMA beginning at the 0.5 mm distance from the defect edge (Figure 3). In addition, the cement volume of the Paste-CPC bone cement was marginally but significantly higher than that of PMMA cement in and directly adjacent to the cement cylinder (Figure 3).

### 3.2. Histology

**Figure 4:**
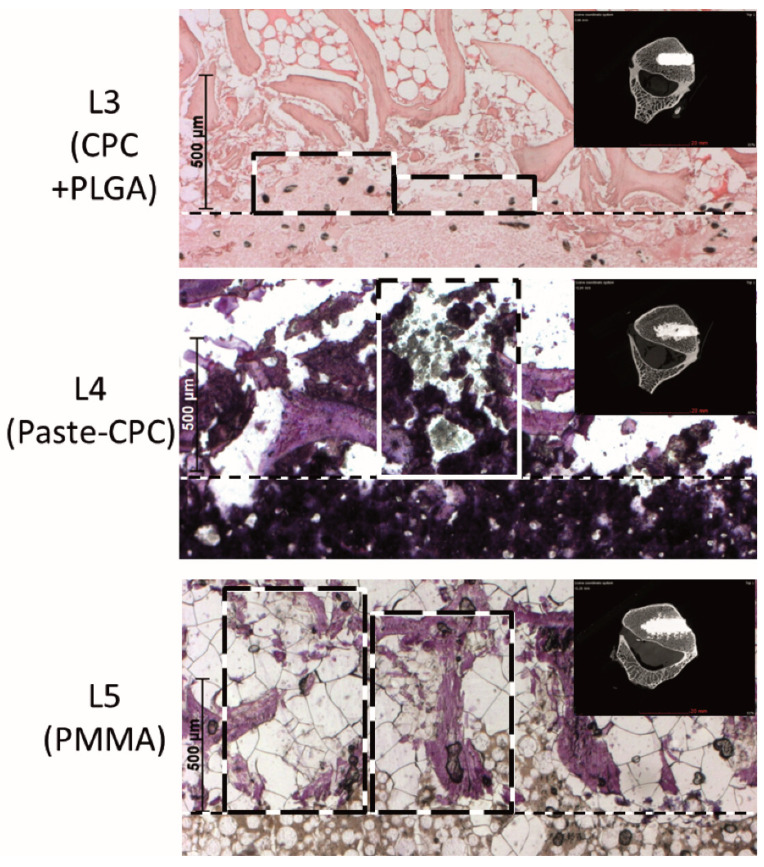
Eosin/hematoxylin (**top**) or hematoxylin staining (**middle** and **bottom**) of representative sections demonstrating the extrusion of CPC + PLGA fibers (**top**), Paste-CPC (**middle**), and PMMA bone cement (**bottom**) into the spongiosa of the ex vivo injected vertebral bodies; inserts display the corresponding micro-computed tomography images; the original defect edge is marked by the dashed line; boxes with dashed lines illustrate regions of cement extrusion, scale bars the distance from the defect edge (500 µm); CPC, calcium phosphate cement; PLGA, poly(L-lactide-co-glycolide); PMMA, polymethylmethacrylate.

The decreased extrusion of the CPC + PLGA in comparison to both Paste-CPC and PMMA bone cement was fully confirmed in histological sections, in which the extrusion of the CPC + PLGA was limited to a distance of approx. 200 µm, whereas the extrusion of Paste-CPC and PMMA exceeded more than 700 to 1000 µm (Figure 4).

### 3.3. Biomechanical Testing

**Figure 5:**
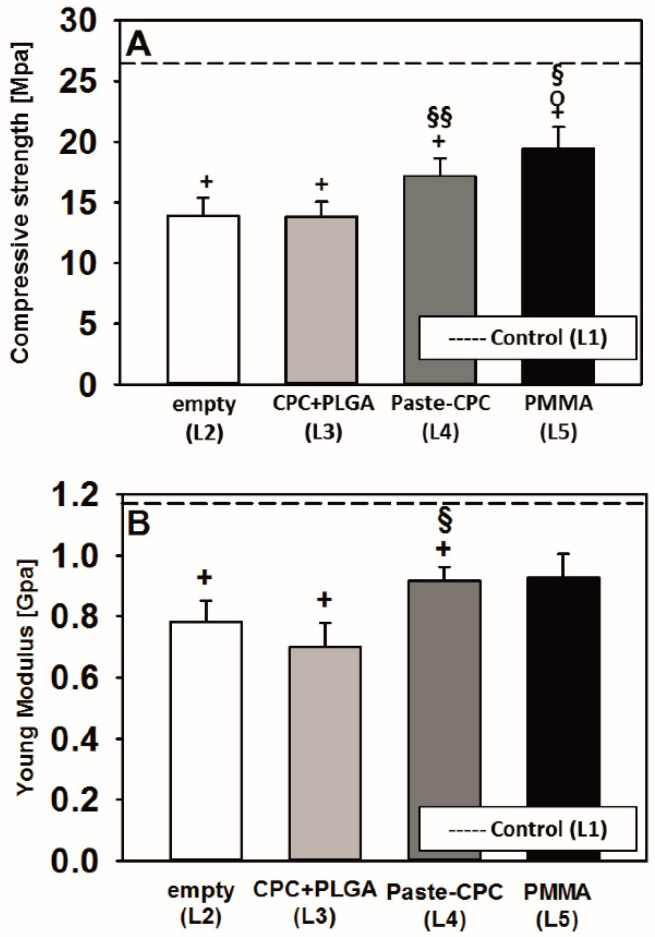
Compressive strength (**A**) and Young’s modulus (**B**) of vertebral spongiosa cylinders following application of CPC + PLGA (L3), Paste-CPC (L4), or PMMA (L5); + *p* ≤ 0.05 versus untouched control (L1); O *p* ≤ 0.05 versus empty defect (L2); § *p* ≤ 0.05, §§ *p* ≤ 0.01 versus CPC + PLGA (L3); mean values of untouched control vertebrae are indicated by dashed lines. CPC, calcium phosphate cement; PLGA, poly(L-lactide-co-glycolide); PMMA, polymethylmethacrylate.

The compressive strength of spongiosa cylinders from all treated vertebral bodies was significantly lower than that of the untouched control (empty defect: −45.3%; CPC + PLGA fibers: −45.7%; Paste-CPC: −32.5%; PMMA: −23.5%; all *p* ≤ 0.05; Figure 5A). However, Paste-CPC showed a significantly higher compressive strength than that of CPC + PLGA, and PMMA showed a significantly higher strength than that of empty defects and CPC + PLGA (Figure 5A).

The Young’s modulus of the spongiosa cylinders from all treated vertebral bodies also exhibited significantly or numerically reduced values in comparison to those of the untouched control (empty defect: −33%; CPC + PLGA: −31%; Paste-CPC: −16%; all *p* ≤ 0.05; PMMA: −9%; Figure 5B). Similarly to the compressive strength, Paste-CPC showed significantly higher values than those of CPC + PLGA (Figure 5B).

## 4. Discussion

In the present ex vivo study with sheep lumbar vertebrae, the extrusion of conventional CPC + PLGA into adjacent spongious bone marrow was significantly lower than for Paste-CPC and PMMA cement. This was demonstrated by micro-CT and validated by histology. Concerning the enhanced intravertebral extrusion of the commercial PMMA cement, the current study confirms our previous studies [17] and extends previous experimental benchmark analyses in artificial porous bodies [25,26].

### 4.1. Extrusion Patterns of Conventional CPC (+ PLGA Fibers) and Oil-Based Paste-CPC

The present report is the first study to show that application of injectable, conventional CPC (± PLGA fibers) for VP decreases the cement extrusion into adjacent spongiosa compared to oil-based Paste-CPC. In addition to frequently reported extraosseous leakage [1,27,28,29], such intraosseous spongiosa extrusion may also foster pulmonary embolism by the entry of cement into basivertebral or segmental vertebral veins [30,31,32,33,34,35]. Cement leakage in human osteoporotic lumbar spines has previously been analyzed, but the data were limited to extrusion into the paravertebral space and spinal canal and failed to address intraosseous leakage and leakage volume quantification [36]. Other authors qualitatively described leakage of PMMA or CPC in almost 50% of injected vertebrae (sometimes into the spinal canal), but a quantitative estimate of the intravertebral leakage was not provided [37]. Lastly, cement extrusion into artificial porous bodies was analyzed (e.g., [25,26]), but quantitation of the **ex vivo intraosseous** extrusion of different bone cements was not provided.

### 4.2. Possible Extrusion Mechanisms

The influence of the following factors on potential cement extrusion mechanisms during VP or KP has been thoroughly addressed in the past: (i) parameters of bone and fractures [26,34,38]; (ii) injection methods [38]; and (iii) bone cement physicochemical features (e.g., viscosity or powder-to-liquid proportion [26,38]). In theory, extrusion may proceed through either a ‘least resistance path leakage’ via intraosseous, low resistance veins or through a ‘pressure sink’ via damaged spongious bone marrow or corticalis [26,39].

The current study provided the following indications for cement extrusion via large veins: (1) conventional CTs showed central basivertebral vessels containing cement in 10% of the CPC + PLGA injections but in the majority of the Paste-CPC (60%) or PMMA injections (80%; data not displayed); (2) micro-CTs displayed venous patterns of either CPC + PLGA (15% of the vertebral bodies), Paste-CPC (75%), or PMMA (90%; compare with Figure 2C,E). However, the different bone cements also leaked through damaged bone marrow channels caused by defect generation (compare with Figure 4). For PMMA, a contribution of high temperature during polymerization and subsequent tissue necrosis can be discussed [40,41]. Thus, leakage through both damaged spongiosa and venous blood vessels must be considered, as supported in the latter case by seminal studies demonstrating the relevance of vertebral veins for the extrusion of bone cement in VP or KP [34,35,42].

The site of injection could also be pivotal for the reported cement leakage based on: (i) the hour-glass shape of the vertebrae [43]; and (ii) the central craniocaudal location of large basivertebral veins (analogous to human vertebral bodies [35]; compare Figure 1 and Figure 2C,E). In this study, the cement was injected into the caudal third of the vertebral body [37,44], a site which is a considerable distance from the large central basivertebral veins. Thus, cement extrusion via these veins may have been limited.

The pressure used for cement application in the present study was subjectively regarded as low by an experienced surgeon, possibly because the cement was injected into a pre-formed hole [25]. Analogous to clinical VP, the target vertebra was completely filled with cement using standardized, commercial fillers and pestles (Medtronic), allowing a direct feedback of the applied force to the surgeon. Such low-pressure vertebral injections are, in general, less likely to induce intra- or extra-vertebral cement leakage [45,46].

The injected cement volume is yet another factor influencing cement leakage [27,47]. While in previous clinical studies, an average of approx. 3 mL of PMMA cement was applied (range of 0.13 to 10.8 mL), only small, low cement volumes could be injected into the present drill hole (Figure 2; right column; approx. 0.27 mL; [17]). Therefore, the present cement volume only amounted to approx. 1.65% of the total vertebral volume, while clinically approx. 9% of this volume were applied and even 24% appeared optimal for pain relief ([17] and references therein). However, even with such small volumes, the differences between the extrusions of the three different cements were clearly significant.

Cement viscosity also critically influences cement leakage during VP or KP ([17] and references therein, [25,26,42]), and PMMA with higher viscosity commonly reduces extravasation [17,25,26,45]. Recommended viscosity for CPC (paste-like) and PMMA (doughy not-sticking state; Kyphon HV-R) was achieved in the current study. Under these conditions, PMMA was more viscous than CPC + PLGA throughout the setting, but it showed lower respective viscosity in the final phase of a shear stress profile simulating the injection process (preliminary rheometer results; data not shown; [48]). Thus, decreased PMMA viscosity during or directly following injection may also represent a factor for enhanced intravertebral leakage. Due to the lack of respective rheometer data for the novel Paste-CPC, a contribution of viscosity differences between Paste-CPC and the other two bone cements to the extrusion behavior in the current study cannot be completely excluded. Indeed, a less pronounced increase in the extrusion force observed previously upon extrusion of the Paste-CPC as compared to a conventional powder/liquid CPC (similar to the CPC + PLGA) from 3 mL Medmix syringes may suggest a lower viscosity of the Paste-CPC, at least in comparison with conventional powder/liquid CPCs [16].

Theoretically, the surfactants contained in the initial composition of the Paste-CPC (CPC powder mixed with 2.5% K_2_HPO_4_ in an oil-based suspension (synthetic short-chain triglyceride Miglyol 812 with 8–12 C saturated fatty acids containing two surface-active agents, i.e., 14.7% (*w*/*w*) castor oil ethoxylate 35 and 4.9% (*w*/*w*) hexadecyl-phosphate)) may have influenced its extrusion behavior by enhancing the interaction with the water-containing environment. However, this initial composition is expectedly strongly hydrophobic and, as a consequence, shows little interaction with 0.9% NaCl within 24 h of incubation; i.e., it retains the tube-like shape without any disintegration and releases only 0.2% of the initial paste-CPC solids into the surrounding medium [16]. Thus, an influence of the two surfactants on the extrusion behavior of the Paste-CPC in the current study is rather unlikely.

### 4.3. Biomechanical Considerations

Paste-CPC or PMMA tendentially or significantly augmented the biomechanical features of spongiosa cylinders when compared to empty defects and/or CPC + PLGA, a result possibly supported by the enhanced interlocking of Paste-CPC or PMMA with the bone trabeculae in the vertebral spongiosa. Despite experimental and quantitative differences, the present study generally confirms previous publications ([17] and references therein). On the other hand, Paste-CPC or PMMA was unable to completely reestablish the compressive strength of untouched control vertebrae, possibly due to either the limited injected volume (1.65% versus 15% to 20% in previous analyses [17,49]) and/or to the brittle character of CPCs [48,50]. The different biomechanical features of CPC + PLGA and PMMA are in contrast to those in previous reports, which have shown no or only numerical differences [17,37,51]. A comparable biomechanical strength of the most commonly used PMMA bone cement and oil-based Paste-CPC may qualify the latter for osteoporotic vertebral body fracture augmentation, with the potential to avoid the cement failure and radiographic loss of correction previously reported for other CPCs [52].

A clear limitation is that the present study did not focus on the relative importance of specific mechanisms of cement extrusion or on directly safety-related in vivo parameters. In addition, there was no comparative analysis of the relative contribution of these mechanisms to the cement extrusion in bones from different parts of the sheep or human body and, thus, to the specific relevance of the extended vertebral vein system for the observed cement extrusion [34,35,42]. A contribution of tissue damage induced by deep-freezing and defrosting of the sheep spine samples to the extrusion pattern was highly unlikely, as histology samples that had undergone the same freezing and defrosting procedure, but were subsequently processed for decalcified and paraffin-embedded sections did not show any indications of such damage (compare with Figure 4, upper panel).

## 5. Conclusions

This is the first study to systematically show a significantly decreased extrusion of conventional CPC (+ fibers) in comparison to Paste-CPC and PMMA cement in the immediate vicinity of the injection channel. Increased extrusion of Paste-CPC could, on the one hand, be favorable for a sufficient biomechanical stabilization of osteoporotic vertebral fractures following VP or KP and a reduction of the risk of cement failure or radiographic loss of correction seen with other CPCs [52]. Similar application and biomechanical properties to those of gold standard PMMA bone cement may qualify the ready-to-use Paste-CPC for spinal surgery. On the other hand, the risk of extrusion into adjacent spongiosa may be diminished by PLGA fiber reinforcement.

Given different biomechanical properties of bones in the human body and the request of the clinical users for improved bone replacement materials, multiple options of tailored bone-filling materials are highly desirable. The present study thus aimed at providing a better background for a rational choice between marketed or newly developed bone replacement materials and to possibly combine the strengths of different technologies.

## Data Availability

The data presented in this study are available on request from the corresponding author.
